# Fostering inclusion in healthcare: a systematic review of collaborative teaching with individuals with disorders of intellectual development

**DOI:** 10.1186/s12909-026-09930-0

**Published:** 2026-07-17

**Authors:** Felix Gorny, Anika Grohmann, Mareike Müller-Cleve, Bavaresco Daniela, Saskia Wirtz, Christan Brandt, Tanja Sappok

**Affiliations:** 1 Universität Bielefeld, Medizinische Fakultät und Universitätsklinikum OWL, Universitätsklinik für Inklusive Medizin, Krankenhaus Mara, Maraweg 21, Bielefeld, 33617 Germany; 2 Bielefeld University, Medical School and University Medical Center OWL, Mara Hospital, Department of Epileptology, Maraweg 21, Bielefeld, 33617 Germany

**Keywords:** Co-teaching, Intellectual disability, Inclusive education, Healthcare training, Educational inclusion

## Abstract

**Background:**

Enhancing healthcare professionals’ knowledge and skills through co-productive educational programs aligns with the UN’s rights for people with disabilities. Collaborative teaching approaches, where individuals with Disorders of Intellectual Development participate as co-teachers, are increasingly recognized as effective strategies for improving healthcare training and fostering inclusion.

**Methods:**

This review examines interventions involving individuals with Disorders of Intellectual Development as co-educators in higher education, focusing on studies published from 2009 to 2024. Inclusion criteria required: Co-teachers with Disorders of Intellectual Development, teaching in higher education, publications after 2009, university or medical school settings. Exclusion criteria eliminated studies without co-teachers with Disorders of Intellectual Development, those involving Disorders of Intellectual Development only as students or patients, and non-university settings. Literature searches were conducted across major databases (PubMed, Cochrane, Psyndex, Web of Science, FIS Bildung, peDOCS, and Google Scholar), between April 25th and November 29th 2024, and articles were screened according to predefined criteria.

**Results:**

Out of 21,327 records identified, 10 met the inclusion criteria, 2 articles were further included for additional consideration. In total, seven empirical studies, three literature reviews, one guideline and one case study were included. Most research focused on the impact of inclusive teaching on students, with some examining the qualification and participation processes for educators with Disorders of Intellectual Development. The evidence supports the value of co-teaching models involving individuals with Disorders of Intellectual Development, highlighting benefits for both students and co-teachers. However, the literature notes that systemic barriers and insufficient structural support can limit the effectiveness and sustainability of such approaches. Standardization among interventions proved insufficient.

**Conclusions:**

Inclusion of co-teachers with Disorders of Intellectual Development is an emerging and important field in higher education as could be shown by this literature review. There is a need for standardized, evidence-based guidelines and adequate institutional resources to support co-teaching involving individuals with Disorders of Intellectual Development in healthcare education. Although inclusive teaching indicates benefits for both, students and educators, their success depends on institutional commitment and overcoming persistent barriers. Ongoing research and policy development are essential to fully realize the potential of co-productive educational models in fostering genuine inclusion and improving healthcare outcomes.

## Introduction

The implementation of the United Nations Convention on the Rights of Persons with Disabilities [1] in Germany has faced relevant criticism. An international audit ranked Germany 35th among the evaluated nations in terms of everyday life implementation [2]. The country received its lowest scores in Education (Art. 24), followed by Health (Art. 25). The 2023 audit identified multiple critical shortcomings, including a lack of concrete improvement measures, insufficient implementation across sectors such as healthcare, inadequate involvement of affected individuals and their organizations, and an absence of systematic data collection regarding the living conditions of people with disabilities. Moreover, the need for regular training of medical staff on human rights, dignity, autonomy, and the specific needs of people with disabilities was emphasized. These findings highlight the urgent necessity for structural reforms and targeted educational initiatives within the healthcare system.

According to the International Classification of Diseases, 11th Revision [3], Disorders of Intellectual Development are characterized by significant limitations in adaptive behavior and intellectual functioning, which originate during the developmental period. Clinical symptoms of Disorders of Intellectual Development include marked impairments in cognition, emotional regulation, and behavior. These symptoms typically reflect underlying dysfunctions in cognitive or biological functioning and developmental processes. Disorders of Intellectual Development is diagnosed along a severity spectrum (mild, moderate, severe, profound) based on the extent of deficits in adaptive functioning [3]. The disorder is considered chronic and may co-occur with additional mental health conditions, including depression, attention-deficit/hyperactivity disorder, and autism spectrum disorder [1–4].

People with disabilities, especially those with neurodevelopmental disorders, encounter numerous barriers and disparities when accessing healthcare systems. Their specific needs (e.g., communication support) are often inadequately addressed by healthcare providers [5]. Consequently, individuals with Disorders of Intellectual Development experience a substantially reduced life expectancy compared with the general population [6, 7].

Molnar et al. argue that persistent stigma, limited disability-specific knowledge and poor communication practices reflect insufficient education about intellectual disability in health curricula and the absence of mandatory disability training for clinicians, and they identify high-quality education as a key lever to reduce these barriers [8]. To enhance healthcare outcomes for this population, it is vital to increase awareness among medical students and professionals regarding their unique healthcare needs, thereby promoting equitable and effective medical care [9].

In certain countries, such as the United Kingdom, and within specific academic disciplines, including social work, concepts of “service user involvement” are already established [10]. The term “service user” refers to individuals who use or have used services such as mental health or disability-related services, regardless of whether these services are currently being accessed or were utilized in the past [11]. The notion of “Involvement” implies that the service user becomes “active in (…) [their] own care and in that of others in similar situations” [11]. Research has demonstrated that this approach contributes to positive changes in students’ attitudes and behaviours towards the relevant target group [12].

In medical education, simulated patients are frequently employed to enhance instruction and improve students’ comprehension and future patient care. Studies [13–15] examining co-teaching initiatives that involve simulated patients with Disorders of Intellectual Development or trained actors have reported positive effects on students’ knowledge and attitudes toward individuals with Disorders of Intellectual Development. However, hesitancy toward these educational approaches has been observed, particularly among senior faculty members, due to limited experience and guidelines for effective implementation [16].

In order to ensure terminological consistency and comparability with existing research, this review employs the ICD 11 category ‘disorders of intellectual development’ when delineating the study population at a diagnostic level. At the same time, person-first formulations are used in the descriptive sections to reflect the inclusive and participatory orientation of the work (e.g. ,individuals with disorders of intellectual development’). This approach acknowledges that diagnostic terminology may be clinically useful while also carrying broader social meanings, especially in participatory and co-produced research. Accordingly, acronyms are avoided as stand-alone descriptors for people, and emphasis is placed on participants’ roles as co-teachers, collaborators, and experts by experience.

In line with broader calls for co-produced education, Molnar et al. advocate that programs for health professionals should be co-designed and co-delivered with people with intellectual disability, who function as co-educators and partners in developing more inclusive, person-centred healthcare [8]. Towle’s taxonomy delineates a continuum of patient involvement in medical education across six progressive levels. Initially, patients are represented abstractly through paper-based or electronic case studies, with no direct interaction. In the subsequent stage, standardized or volunteer patients participate in clinical settings with scripted roles designed to achieve specific learning objectives. As involvement deepens, patients begin to share personal experiences within structured curricula, granting increased autonomy. At a more advanced stage, patients act as patient-teachers, actively involved in instruction and student evaluation. This evolves further when patients become equal partners in educational processes—participating in teaching, assessment, and curriculum development. At the highest level, patients maintain sustained roles as educators and additionally participate at the institutional level, influencing governance and policies regarding health professional education [17].

Particular attention should be directed toward disciplines in which interaction between individuals with and without Disorders of Intellectual Development constitutes an inherent component of vocational practice, as is the case in medicine [18]. Since patient involvement represents a key strategy for promote inclusive healthcare in the long term, it is essential to assess the current state of patient-involved teaching approaches as reflected in both German and English academic literatures. This review aims to contribute to this understanding by analyzing existing evidence on the involvement of individuals with Disorders of Intellectual Development in higher education, specifically in the role of co-teachers.

## Methods

### Search strategy

The research strategy followed the principles of systematic literature searching and reporting in accordance with PRISMA [19]. A comprehensive search strategy was developed using relevant keywords and subject headings related to disorders of intellectual disability, co-teaching, inclusive education, and healthcare training (”Intellectual disability” AND ”Inclusive education” OR ”Healthcare training” AND ”Co-teaching” along with Medical Subject Headings (MeSH terms) (see Table 1). To improve reproducibility, the Boolean search syntax for e.g. the PubMed search was as follows: (“cognitive disorder“[Title/Abstract] OR “intellectual disabilit*“[Title/Abstract] OR “cognitive impairment“[Title/Abstract] OR “intellectual development disorder“[Title/Abstract] OR “disorder of intellectual development“[Title/Abstract] OR “disorders of intellectual development“[Title/Abstract] OR “learning disability“[Title/Abstract] OR “expert* by experience“[Title/Abstract] OR “service user involvement“[Title/Abstract] OR “Partizipativ*“[Title/Abstract] OR “kognitive Beeinträchtigung“[Title/Abstract] OR “Intelligenzminderung“[Title/Abstract] OR “geistige Behinderung“[Title/Abstract] OR “Lernbehinderung“[Title/Abstract] OR “Lernschwierigkeit“[Title/Abstract] OR “Bildungsfachkraft“[Title/Abstract]) AND (“co-teaching“[Title/Abstract] OR “co-teacher*“[Title/Abstract] OR “co-educator“[Title/Abstract] OR “co educator“[Title/Abstract] OR “inclusive teaching assistant“[Title/Abstract] OR “co-instructor“[Title/Abstract] OR “patient-teacher“[Title/Abstract] OR “education*“[Title/Abstract] OR “higher education“[Title/Abstract] OR “university“[Title/Abstract] OR “teaching“[Title/Abstract] OR “Lehre“[Title/Abstract] OR “Hochschullehre“[Title/Abstract] OR “Universität“[Title/Abstract] OR “Hochschulbildung“[Title/Abstract]). The included sources were selected to ensure broad coverage of biomedical, psychological, educational, and interdisciplinary literature. PubMed and Cochrane databases were used to capture health and intervention-related evidence, Psyndex and Web of Science to include psychological and cross-disciplinary studies, and FIS Bildung and peDOCS to identify German-language educational literature that might otherwise be underrepresented. The search strategy illustrated above was tailored to the specific syntax and input requirements of each database and successively executed in all selected information sources. In addition, Google Scholar and related sources were searched by systematically combining the operators specified in the search string above in different constellations. This iterative approach allowed us to explore alternative formulations of key concepts and to identify further relevant publications that might not have been captured by the primary database queries. All resulting records were screened using the same eligibility criteria as for the database search.

### Eligibility criteria

The following databases and sources were searched: PubMed, Cochrane Library, Web of Science, Psyndex, Congress Abstracts, FIS Bildung, peDOCS, and Google Scholar. The search covered studies published from 2009 to 2024, was restricted to human studies, and applied no language restrictions. In addition, the reference lists of all included studies were screened to identify further relevant publications.

The included sources were selected to ensure broad coverage of biomedical, psychological, educational, and interdisciplinary literature. PubMed and Cochrane were used to capture health and intervention-related evidence, Psyndex and Web of Science to include psychological and cross-disciplinary studies, and FIS Bildung and peDOCS to identify German-language educational literature that might otherwise be underrepresented. Google Scholar and Congress Abstracts were used as supplementary sources for grey literature and additional context.

### Criteria for selection of studies

This review focuses on literature describing interventions involving individuals with Disorders of Intellectual Development participating in teaching roles within higher education or medical training.

Studies were included if: (1) the co-teacher was an individual with a Disorder of Intellectual Development; (2) the person participated in a lecturer or co-teacher role; (3) the publication appeared after 2009; and (4) the intervention took place in medical training or university-level education.

Studies were excluded if: (1) co-teachers were not individuals with Disorders of Intellectual Development; (2) individuals with Disorders of Intellectual Development were only the target group of teaching rather than co-teachers; or (3) they participated only as simulated patients.

For the purposes of this review, *co-teaching* was operationalised as the structured participation of an individual with a Disorder of Intellectual Development in the planning, delivery, and/or facilitation of teaching sessions in higher education or healthcare-related training, in collaboration with academic staff. Co-teaching required an active instructional role that went beyond being a guest speaker, simulated patient, or case example. Eligible roles included, for example, co-lecturer, educational specialist, expert by experience, inclusive teaching assistant, or similar designations, provided that the person with a Disorder of Intellectual Development contributed substantively to teaching activities (e.g. presenting content, co-facilitating discussions, or participating in assessment). Studies in which individuals with a Disorder of Intellectual Development were only recipients of teaching, were involved solely as simulated patients, or were present without a defined instructional function were excluded.

Outcomes of the studies were description of the experiences and / or results existing in the planning and implementation process of co-teaching activities.

### Screening and study selection

Two reviewers (Gorny and Grohmann) independently screened titles and abstracts according to the predefined criteria. Disagreements were resolved through consultation with a third reviewer (Müller-Cleve). The screening process was conducted in Citavi.

**Fig. 1:**
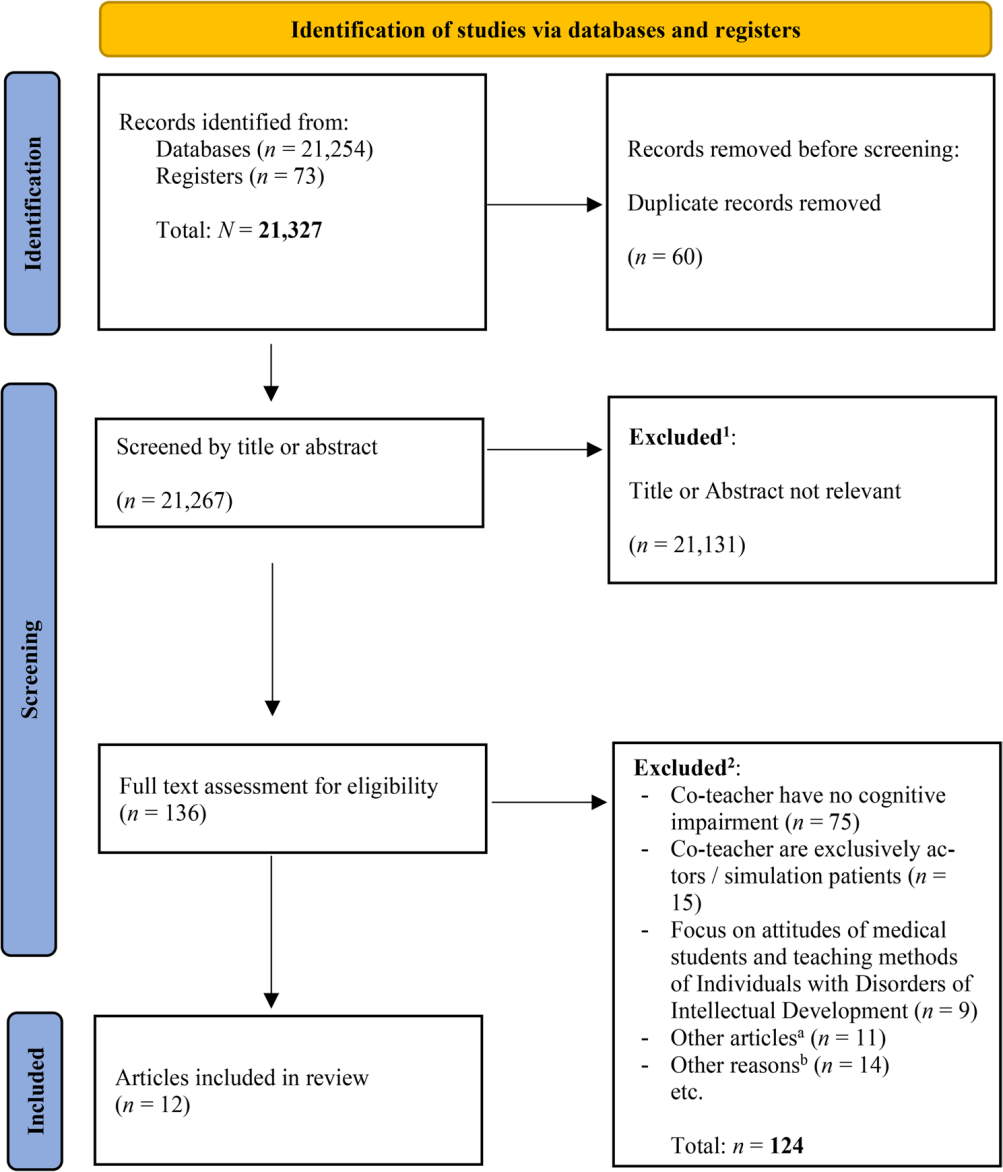
Provides the PRISMA flow diagram of data selection process resulting in the finally included number of articles. Notes: (1) Most records were excluded during the initial screening phase due to titles or descriptions indicating a lack of relevance to the review’s objectives. (2) Further exclusions during later screening stages are detailed along with specific reasons for exclusion. (3) (a) Records categorized as “other articles” primarily consisted of project reports lacking associated studies or scientific evaluation. 4. (b) Records excluded for “other reasons” were topically relevant but did not meet the formal inclusion criteria, instead providing general context

The PRISMA flow diagram in Fig. 1 illustrates the study selection process. A total of 21,327 records were identified, 60 duplicates were removed, 21,267 records were screened by title and abstract, 136 full texts were assessed for eligibility, and 12 items were included in the final synthesis.

### Data extraction

Two investigators (Grohmann and Müller-Cleve) independently extracted data from the included primary studies. Extracted information included country of origin, study design, research aim, methodology, and main findings. Disagreements were resolved through discussion with a third reviewer (Gorny).

Meta-analysis was not feasible, as the included studies did not report comparable quantitative outcomes. Therefore, the data were synthesized and presented descriptively and organized through thematic analysis.

Six a priori analytic categories guided the synthesis (cf. Table 1): [1] qualification of co-teachers and planning experiences [2], mode of integration into educational activities [3], experiences during implementation [4], impact on students [5], impact on co-teachers, and [6] effects on faculty members.

**Table 1:** Keywords sorted by search components, inclusion and exclusion criteria

	Inclusion Criteria	Search Terms	Exclusion Criteria
Population	Individuals with Disorders of Intellectual Development	Cognitive disorder OR intellectual disabilit* OR cognitive impairment OR intellectual development disorder OR disorder of intellectual development OR expert* by experience OR service user involvement. Partizipativ* OR kognitive Beeinträchtigung OR Intelligenzminderung OR geistige Behinderung OR Lernbehinderung OR Lernschwierigkeit OR Bildungsfachkraft.	Individuals without Disorders of Intellectual Development
Intervention	Individuals with Disorders of Intellectual Development act as co-teachers in higher education/medical training and are involved in teaching activities. Published after 2009.	Education* OR higher education OR university OR teaching OR Lehre OR Hochschullehre OR Universität OR Hochschulbildung	Individuals with Disorders of Intellectual Development are the target group of inclusive teaching activities or act as simulated patients and not as co-teachers (based on Towle’s taxonomy).

Because terminology for participatory teaching roles varies across countries and disciplines, studies were not selected on the basis of specific job titles alone. Instead, inclusion was determined by the underlying function of the role, that is, whether the individual with a Disorder of Intellectual Development fulfilled the operational co-teaching criteria described above.

### Approach to quality assessment

Given the exploratory aim of this review and the heterogeneity of the included publication types, no formal quality appraisal was undertaken. The purpose of the review was not to determine the effectiveness of a narrowly defined intervention on the basis of high-quality comparative studies, but rather to capture the available literature on the involvement of individuals with disorders of intellectual development as co-teachers in higher education and healthcare-related teaching as comprehensively as possible. Because the included literature comprised methodologically diverse empirical studies as well as reviews, a guideline, and a case study, the use of a single formal appraisal tool was considered to have limited utility for the present review question. In addition, much of the available literature in this field is exploratory, descriptive, and variably reported. Applying formal quality thresholds would therefore likely have reduced the breadth of the synthesis without substantially altering the overall conclusion that the evidence base is still limited and heterogeneous. Instead, methodological characteristics and limitations of the included publications were considered throughout the descriptive synthesis and are addressed explicitly in the Discussion and Limitations sections.

The PRISMA flow diagram [20] in Fig. 1 illustrates the data selection process, ultimately resulting in the inclusion of 12 studies in this review.

### Result assessment and categorization

Additional studies were identified through references found during the initial literature screening. A thematic analysis was conducted to answer the research question and systematically examine international experiences and outcomes related to the participation of individuals with Disorders of Intellectual Development as co-teachers in university-level teaching. The following analytic categories were defined to guide data extraction and synthesis:

1. Qualifications of co-teachers and experiences during the planning phase of implementation.
2. Mode of integration of co-teachers with Disorders of Intellectual Development into educational activities.
3. Experiences encountered during the implementation phase of co-teaching.
4. Impact on student participants.
5. Impact on co-teachers with Disorders of Intellectual Development.
6. Effects on faculty members.

*Category 1* pertains to the planning processes and qualification measures undertaken for the inclusion of individuals with Disorders of Intellectual Development in teaching roles, encompassing both facilitators and barriers encountered, as well as the presence or absence of formal training.

*Category 2* describes how co-teachers with Disorders of Intellectual Development were integrated into educational settings, outlining the course types and instructional formats used.

*Category 3* encompasses the range of experiences observed during the active implementation of co-teaching, including both positive outcomes and challenges.

*Categories 4*, *5*, and *6* focus on the respective effects and experiences reported by students, co-teachers with Disorders of Intellectual Development, and faculty members within the context of these educational interventions.

## Results

The literature reviewed yielded heterogeneous findings across the predefined thematic categories of the content analysis: Of the ten studies included, eight reported findings that aligned with the established categorical framework, whereas two studies [6, 21] did not provide data relevant to any of these categories. In addition, two articles describing project-based interventions were identified [22, 23]. Although these did not meet the methodological criteria for inclusion as formal studies, they offered valuable insights into the research question and were therefore incorporated into the thematic analysis. Accordingly, these works are included in the results presented herein.

Seven primary studies were included, originating from Germany (*n* = 2), the United Kingdom (*n* = 1), Australia (*n* = 1), Norway (*n* = 1), Ireland (*n* = 1), and Spain (*n* = 1). Additionally, three literature reviews were included, all conducted in the United Kingdom (*n* = 3). Two studies [24, 25] utilized pre- and post-intervention assessments to evaluate the impact of educational programs on student attitudes. In particular, Mau et al. [24] investigated changes in attitudes toward inclusion and students’ self-efficacy expectations.

The study examined 35 pre-service teachers, currently in training, (66% women; mean age 23.48, SD 3.13) participating in a semester-long seminar on intellectual disability and inclusion, assessing attitudes with the MTAI-D and self-efficacy with the “Lehrer-Selbstwirksamkeitsskala” (engl. “Teacher Self-Efficacy Scale”) alongside additional background and a global change item. While 56% of students reported a perceived change in attitudes (mean 3.65/6), no statistically significant pre–post differences were found across inclusion-related attitudes or self-efficacy scales (all $\:p>.05$).

Two studies [26, 27] examined the perceived impact of educational interventions delivered by individuals with Disorders of Intellectual Development from the perspective of student participants. Rodríguez Herrero et al. [28] additionally explored the experiences of co-teachers and faculty members. Another study [29] focused on both the process of integrating individuals with Disorders of Intellectual Development into academic courses and the effects of their participation on students. Mevold et al. [30] aimed to evaluate innovative instructional tools and their influence on medical students in the context of Disorders of Intellectual Development. One review [21] analyzed medical students’ attitudes toward individuals with Disorders of Intellectual Development, while the remaining reviews [6, 31], while the remaining studies assessed the effectiveness of educational interventions involving individuals with Disorders of Intellectual Development and their impact on medical students. One publication [23] presented a case study and subsequent evaluation of an educational intervention, providing insights into the implementation process and outcomes. Another source [22] presented a guideline for the qualification and integration of co-teachers, contributing relevant context to the research inquiry.

**Fig. 2:**
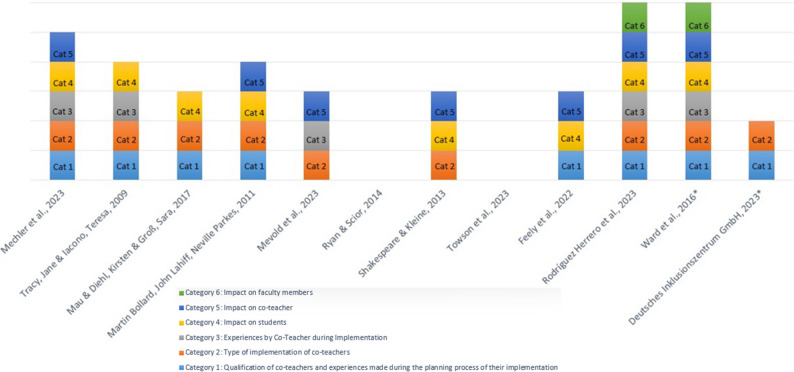
Overview of content assessment categories and corresponding literature. The figure summarizes the six analytic categories used to structure the synthesis of studies on co-teaching with individuals with Disorders of Intellectual Development: (1) qualification and preparatory experiences of co-teachers, (2) mode of qualification and integration of co-teachers into educational activities, (3) experiences during implementation of co-teaching, and (4–6) reported impacts on students, co-teachers, and faculty members. Each included publication is mapped to one or more categories, illustrating which aspects of the co-teaching process are well documented and where evidence remains sparse. Studies marked with an asterisk (*) were identified in a subsequent search step and, although not part of the primary empirical dataset, contribute additional contextual information to one or more content categories

Table 2 provides a comprehensive synthesis of studies and reviews that examine the characteristics, implementation strategies, and outcomes of educational interventions involving individuals with Disorders of Intellectual Development or learning disabilities as educators. Particular emphasis is placed on their impact on learners’ attitudes, knowledge acquisition, and professional development across diverse international settings. A comprehensive summary of the reviewed literature, organized according to content assessment categories, is presented in Fig. 2. Most articles described the qualification of the co-teachers, the mode of integration, and the impact on students. While the effects on co-teachers was reported in 7/12 articles, the effects on faculty was rarely examined (2/12).

**Table 2:** Overview of educational interventions involving educators with disorders of intellectual development and their impact on learners

Study / review number	Reference	Design^b^ & Analysis methods	Sample size	Aim / question^c^	Role of individual with Disorder of Intellectual Development	Key findings
1	[27]	Design: Qualitativ (Online survey, focus groups, expert interviews) with teaching students. Analysis Methods: Qualitative content analysis	*N* = 78 (Students only; Online survey: *n* = 62 Focus groups: *n* = 13 Expert interviews: *n* = 5)	To highlight the impact of educational offers by co-teachers on teaching students from the students’ point of view	Role: “Bildungsfachkräfte” (BFK); engl.: “Educational Experts“ Educational experts with lived experience of disability (BFK) are integrated into higher education teaching following a three-year qualification program, subsequent to their prior employment in sheltered workshops for people with disabilities. Within educational formats, BFK share their experiential knowledge and relate their perspectives to key concepts such as participation and accessibility. In doing so, they provide insights into their lived environments and enable participants to engage in direct dialogue with them.	Effects of inclusive teaching on students; three main categories: 1. “interpersonal skills (e. g., perspective taking), 2. perceptions on inclusion and persons with disabilities (e.g., self-reflection), 3. Professional knowledge (e. g., behavioural intention as a teacher)” (Mechler et al. 2023, S. 198)
2	[25]	Design: Quantitative (Pre- and Post Tests with medical students based on „The interaction disabled persons scale” (IDP) Analysis Methods: SPSS v11, repeated measures t-test (1-tailed)	*N* = 128	To evaluate changes in the attitudes of medical students towards people with developmental disabilities after participating in a communication skills training.	Role: Tutors wih intellectual disability (ID). Tutors with ID engaged in direct interaction with students following a structured teaching session. This session comprised an initial lecture providing a thematic overview of intellectual disability, followed by facilitated contact with tutors with ID, and concluded with a guided communication exercise. Accordingly, tutors with ID constituted an integral component of the intervention, which was evaluated using a pre–post study design.	The study demonstrates a small but statistically significant improvement in medical students’ comfort with interacting with people with developmental disabilities after a single 3-hour communication skills session, as measured on the Interaction with Disabled Persons Scale (IDP) and supplemented by qualitative student evaluations. The primary outcome was change in self‑reported discomfort in interactions with disabled persons, assessed with the IDP before and immediately after the session.DP total scores can range from 20 to 120, with higher scores indicating greater discomfort in social interaction. In this cohort of 128 fourth‑year medical students, mean IDP scores decreased from 73.7 (SD 10.6) pre‑session to 70.41 (SD 10.2) post‑session, indicating reduced discomfort. A one‑tailed repeated‑measures t‑test yielded a statistically significant result, t(127) = 5.07, *p* < .001, confirming that the observed reduction in scores is unlikely to be due to chance. Although effect sizes are not explicitly reported, the mean change of approximately 3.3 points on a 20–120 scale, relative to the baseline standard deviation of about 10.6, corresponds to a small within-subject standardized mean difference (roughly one-third of a standard deviation), suggesting a modest but measurable attitudinal shift at group level. IDP has previously demonstrated acceptable internal consistency (Cronbach’s alpha 0.74–0.86) and a six-factor structure, but they did not re-estimate reliability or factor structure in this sample.
3	[24]	Design: Quantitative (Pre- and Post Questionnaire, German version of „My Thinking about Inclusion (MTAI-D)) Analysis Methods: IBM statistics 22, t-Test, ANOVA	*N* = 45	To evaluate the impact of courses given by Individuals with Disorders of Intellectual Development on teaching students attitudes towards inclusion and their self-effectiveness.	Role: Educational Professionals In the seminar “Nothing About Us Without Us – People with Disabilities as Experts in Their Own Right,” educational professionals from the Inclusive Education project engage in practical encounters with students. Two of the six educational professionals independently and alternately conduct the seminar, contributing their expertise to academic teaching. They present the “Inclusive Education” project, illustrate the life trajectories and everyday experiences of people with disabilities, and engage students in discussions about personal experiences, interests, aspirations, their professional work, and living situations.	The study reports that participation in a semester-long seminar taught by educational specialists with intellectual disabilities is associated with high self-efficacy ratings and some item-level, but not scale-level, statistically significant changes in attitudes towards inclusion among pre-service teachers. The study used a pre‑post design with two measurement points within a single group of Bachelor‑level pre‑service teachers enrolled in the compulsory module “Heterogenität – Umgang mit Differenz” at Europa‑Universität Flensburg. The analytic sample comprised *N* = 35 students (66% women), aged 20–39 years (mean 23.48, SD 3.13), on average in the 4th semester (mean 3.93, SD 1.82). Attitudes towards inclusion were measured using the German version of “My Thinking about Inclusion” (MTAI‑D), which comprises three Likert‑type subscales: Core perspectives, Expected outcomes, and Classroom practices (19 items, 1–5 response format, higher values indicating stronger agreement). Teacher self‑efficacy was measured using Schwarzer & Schmitz’s 10‑item Lehrer‑Selbstwirksamkeitsskala (1–4 response format, higher values indicating greater self‑efficacy). Additional items captured previous contact with people with disabilities, prior inclusion‑related courses, and a single global item (“Hat sich Ihre Einstellung zur Inklusion … verändert?”) rated on a 6‑point scale. In response to the global change item, students reported a mean of 3.65 (SD 1.39) on a 6‑point scale, indicating that, on average, they “rather” agreed that their attitudes towards inclusion had changed through the seminar. 56% of respondents stated that their attitude had changed. For all scales, there were no statistically significant pre‑post differences at the scale level (paired‑samples t‑tests, *p* > .05 for all). The reported means (M) and standard deviations (SD) are: Expected outcomes (attitudes regarding learning gains of pupils with special needs in inclusive settings): T1: M = 3.28, SD = 0.67 T2: M = 3.41, SD = 0.49 Slight shift from “partly agree” towards “largely agree”, but not statistically significant at scale level. Classroom practices (perceptions of classroom impact and demands): T1: M = 2.37, SD = 0.49 T2: M = 2.29, SD = 0.54 Means remain clearly below the neutral midpoint (3.0), indicating rather negative expectations regarding inclusive classroom management; change not significant. Core perspectives (fundamental stance towards inclusion): T1: M = 3.65, SD = 0.46 T2: M = 3.73, SD = 0.51 Both time points above the neutral midpoint, indicating generally positive core attitudes; change not significant. Teacher self‑efficacy: T1: M = 3.18, SD = 0.36 T2: M = 3.14, SD = 0.34 Means close to “rather applies”, indicating overall high self‑efficacy; no significant scale‑level change. Paired‑samples t‑tests (two related samples) were calculated for each of the four main scales (Core perspectives, Expected outcomes, Classroom practices, self‑efficacy) between T1 and T2. None of these t‑tests reached statistical significance at α = 0.05; the authors explicitly state that the changes in scale means were not significant.
4	[29]	Design: Qualitative analysis of findings and discussion from two projects involving students and people with learning disabilities in a 3-year learning disability nursing programme. Project 1: questionnaire Project 2: Focus groups (students and people with Disorders of Intellectual Development) to evaluate the involvement of people with Disorders of Intellectual Development in educational activities Analysis Methods: EPICURE as a qualitative analysis framework	Project 1: questionnaire (*n* = 29, 29% response rate)	To illustrate a qualitative evaluation of including people with Disorders of Intellectual Development in educational courses in context of a 3-year learning disability nursing programme.	Role: Service Users (SU) Service users contribute to developing a faculty-wide service user strategy and are expected to represent as wide a diversity as possible within the learning-disability population. They help to plan and evaluate the course via workshops and recorded (video) input, thus influencing content and pedagogical design. Commissioning of courses: Course development and commissioning are explicitly driven by, and responsive to, the aspirations and wishes of service users through formal consultation processes. This positions people with learning disabilities as agenda‑setters rather than passive consultees. Marketing & publicity: Service users are “willingly and positively” involved in endorsing and marketing courses, indicating an ambassadorial role that supports public representation of the programme. Recruitment and admissions: Service users participate as key members of staff and student interview panels and share in decision‑making, embedding their perspectives into selection processes. Curriculum delivery: Service users teach students directly, for example through introductory “getting to know you” sessions, “sharing knowledge about health” sessions, and activities in a mental health in learning disability module. Assessment: Service Users are involved in practice assessment (e.g. poster evaluations and judging students’ abilities to assess aspects of health and communication), and they also participate in individual debriefings and more formal end‑of‑year feedback sessions for evaluation.	Planning to embed people with Disorders of Intellectual Development in educational activities requires more time, investment and development to find a way that fits to the ability of people with Disorders of Intellectual Development (in context with recruitment, teaching, learning, assessment) The involvement of people with Disorders of Intellectual Development leads to a deeper understanding and learning for students. Benefits also for involved people with Disorders of Intellectual Development.
5	[30]	Design: Qualitative (semi-structured interviews) Analysis Methods: Thematic analysis: Malterud’s systematic text condensation method	Lecturer with Disorders of Intellectual Development (*n* = 6)	To focus the experiences of lecturers with Disoders of Intellectual Development in higher education rather than the experiences of students being taught by people with intellectual disabilities.	Role: Individuals/Lecturers with Intellectual Disabilities (ID) All six individuals are formally employed as lecturers in a health and social education program at a Norwegian university, with employment contracts and payment on an equal footing with other external lecturers. Their specific assignment is to teach about their own experiences of living with “light or moderate cognitive impairment,” presenting life stories as service recipients and thereby functioning as “experts on their own lives.” They lecture for 1–2 h, once or twice a year, mainly in ordinary classroom teaching; another teacher may be present if the lecturer explicitly wants support.	Mainly positive experiences regarding their feelings of having social relations, learning new things and experience self-determination.
6	[21]	Design: Literature review Analysis Methods: Electronic search (Embase, Ovid MEDLINE(R), PsycINFO, Scopus, Web of Science (published after 2013).	24 studies	To enhance the understanding of medical students’ attitudes towards Individuals with Disorders of Intellectual Development.	Role: Target Group/People with Intellectual Disabilities (ID)/Patients/Service Users (SU). Structually central, yet analytically indirect group.	Results suggest that students’ attitudes changed after various interventions
7	[31]	Design: Literature review Analysis Methods: Electronic search (MedLine, English and French)	48 papers	To review what has been trialled and, if possible, the outcomes.	Role: People with ID/Persons with Disabilites (PWD) are regarded as teachers, workshop facilitators, standardized patients, community hosts, and recipients of in-service staff training.	To meet people with Disorders of Intellectual Development had the most positive change in attitudes
8	[6]	Design: Scoping review Analysis Methods: Electronic search (PUBMED, ERIC, Scopus, Web of science, after 1980) and grey literature	16 studies	To evaluate the educational interventions in context of intellectual disabilities and their effects.	Role: People with intellectual disabilities (ID) as experts in lived experience and educator roles. Across the 16 included studies, individuals with intellectual disabilities are mainly positioned as patients, simulated or standardised patients, and recipients of care, whose presence is intended to improve medical students’ knowledge, attitudes, communication, and clinical skills	Most studies involved Individuals with Disorders of Intellectual Development as living input in different ways. Different learning outcomes and difficult to evaluate the effect,
9	[26]	Design: Qualitative Analysis Methods: Thematic analysis (Braun and Clarke)	Questionnaires (*n* = 18) and focus groups (*n* = 4) with students	To analyse the experiences of social work students after participating in a course, designed and delivered by Individuals with Disorders of Intellectual Development.	Role: Adults with intellectual disabilities/people with intellectual disabilities (ID) as lecturers, tutors, and assessors. They are planning and delivering teaching sessions, interacting directly with students, and participating in the grading or evaluative process, rather than appearing only as guest speakers.	Positive impact on students’ empathy and increased feeling of comfort in interacting with Individuals with Disorders of Intellectual Development.
10	[28]	Design: Qualitative & Phenomenological (Interviews & Focus group with teachers with and without Disorders of Intellectual Development and students) Analysis Methods: Content analysis (MAXQDA 2022)	Interviews with co-teachers (*n* = 2) interview with teacher (*n* = 1) focusgroup with students (*n* = 3)	Understanding and analysing the perception of co-teaching activities from different point of views.	Role: Teachers with Intellectual Disabilities (ID) The central role is as university teachers and co-teachers. These teachers participate in joint planning of the module, meeting weekly with the tenured teacher to design sessions, select content, and refine teaching approaches, with the authors emphasising the evolution from a more rigid to a more flexible, co-constructed planning process. In classroom delivery, the tenured teacher primarily provides theoretical input, while the teachers with intellectual disabilities contribute experiential and autobiographical teaching, including accounts of their schooling (special vs. regular settings), employment, and preferred forms of inclusive classroom practice. They assume substantial pedagogical responsibility, leading sessions, inviting other “inclusive teachers,” stimulating discussion, and modelling inclusive practice; the paper notes that sessions in which they took more responsibility had the greatest reported impact on students.	Positive impact on personal & professional competences of the three teachers. Students learnt a lot about communication and their prejudices in terms of disability and inclusion.
11^d^	[23]	Design: Qualitative case study of an inclusive teaching initiative in qualifying social work education, informed by literature review on service user involvement and inclusion of people with profound and multiple learning disabilities Analysis Methods: Descriptive quantitative analysis of student self‑ratings (Likert scales) plus thematic analysis of open‑ended questionnaire responses; reflective qualitative analysis of discussions with Christian and his supporters (auto/biographical, auto‑ethnographic elements).	40 social work students completed evaluation questionnaires across three deliveries of a two‑day elective; 15–20 students per cohort; one man with profound and multiple learning disabilities (Christian) plus members of his circle of support involved as teaching team	To examine how people with profound and multiple learning disabilities, who are often excluded from service user involvement, can be meaningfully and inclusively involved in social work education, and what impact this has on students, the individual and his supporters	The man with profound and multiple learning disabilities is employed and commissioned as a visiting lecturer leading a two-day elective on working with people with learning disabilities; he designs and delivers teaching (with support), uses his own life story and materials (diaries, photos, videos) as auto-ethnographic content, and acts as an expert by experience whose presence, communication and relationships are central to students’ learning	Students reported increased self‑rated knowledge about working with people with learning disabilities, especially those without previous experience, and highlighted experiential learning about communication, personalisation, family perspectives, and challenging assumptions about capability. The initiative demonstrated that attitudes and low expectations are major barriers to inclusive involvement, and that employing a person with profound and multiple learning disabilities as a lecturer can subvert stereotypes, expand his social roles and networks, and promote recognition and parity of participation. At the same time, the paper notes practical, emotional and organisational challenges (room allocation, health fluctuations, emotional labour, risk of over‑burdening) and argues for careful, justice‑oriented development of inclusive involvement rather than tokenistic or romanticised participation.
12^d^	[22]	Design. Conceptual and practice-oriented guideline describing the development, implementation, and sustainable institutionalisation of a three-year full-time qualification programme for “educational specialists” with prior employment in sheltered workshops, preparing them for work at universities. Analysis Methods: Not primarily an empirical research study; synthesises conceptual foundations, curriculum and module descriptions, implementation experiences, and practice examples. Linked projects typically use mixed-methods evaluation (quantitative and qualitative data on effects for students, academic staff, and educational specialists)	No single defined study sample; the guide addresses adults with intellectual disabilities / learning difficulties who previously worked in sheltered workshops and are trained as educational specialists. On this basis, several cohorts (approximately 6–8 participants each) have completed the qualification at different universities.	To describe aims, content, organisational structures, and conditions of the programme “From sheltered workshop to university”: how adults with intellectual disabilities and workshop experience can be qualified and sustainably established as educational specialists in higher education in order to contribute lived experience of inclusion and exclusion to teaching and to open up new, non-segregated employment pathways.	Adults with intellectual disabilities (often described in practice as people with learning difficulties) and prior sheltered workshop experience are trained as “educational specialists” and, after completing the three-year full-time programme, are employed by universities. They act as experts by experience, co-design and co-deliver teaching (e.g. on inclusion, participation, and everyday life with disability), use biographical material in teaching, work in co-teaching tandems with academic staff, and function as change agents for inclusive institutional development.	The guide reports that the qualification is structured in several modules (e.g. inclusion and participation, biographical learning, methods of educational work) and systematically integrates theory, practice placements, and psychosocial support. On the basis of this model, multiple universities have implemented qualification cohorts and subsequently employed educational specialists as salaried teaching staff, which is associated with empowerment effects for the specialists (enhanced self-efficacy, social recognition, independent income) and with educational benefits for students and staff (reduction of stereotypes, increased competence in inclusion and disability, exposure to first-person perspectives on barriers and participation). The guide also targets sustainable structural anchoring (e.g. cooperation agreements, long-term funding, involvement in committees) and conceptualises educational specialists as permanent actors in inclusive higher education development.

a Seven primary studies included: Germany (*n* = 2), UK (*n* = 1), Australia (*n* = 1), Norway (*n* = 1), Ireland (*n* = 1), Spain (*n* = 1). Three literature reviews were conducted in the UK (*n* = 3)

b Studies 2 and 3 employed pre- and post-intervention assessments to evaluate changes in student attitudes; Study 3 specifically addressed attitudes toward inclusion and self-efficacy

c Studies 1 and 9 assessed the impact of educational interventions delivered by individuals with Disorders of Intellectual Development from the student perspective. Study 10 reported experiences of co-teachers and faculty. Study 4 evaluated both the process of inclusion and student outcomes. Study 5 investigated novel teaching tools and their effects on medical students in the context of Disorders of Intellectual Development. Review 6 examined medical students’ attitudes toward Individuals with Disorders of Intellectual Development; Reviews 7 and 8 evaluated educational interventions involving Individuals with Disorders of Intellectual Development and their impact on medical students

d Two project articles excluded by study design but relevant to the research question are listed separately. Article 11 is a case study with teaching evaluation on intervention implementation and outcomes. Article 12 provides guidelines for qualifying and establishing co-teachers

### Qualification of co-teachers and experiences during the planning process of their implementation

Data on the qualifications of co-teachers and the planning of their integration were limited for category 1 (Qualification of co-teachers and experiences during the planning process of their implementation). Two studies reported a three-year training program for co-teachers [24, 27]. While Mau et al. [24] described lecturers being qualified as educational specialists through formal training alone, Mechler et al. [27] specified their qualification process as the completion of a three-year qualification following employment in sheltered workshops, and active participation in higher education for several years, where experiential knowledge could be shared with students. Another study noted that co-teachers had no formal training [28]. Several authors [28, 29] stressed that additional time and resources were required in the planning phase to support effective curriculum development and implementation. They suggested that inadequate investment in these areas could undermine the effectiveness of user involvement, not due to the concept itself, but because of insufficient support structures [29].

**Fig. 3:**
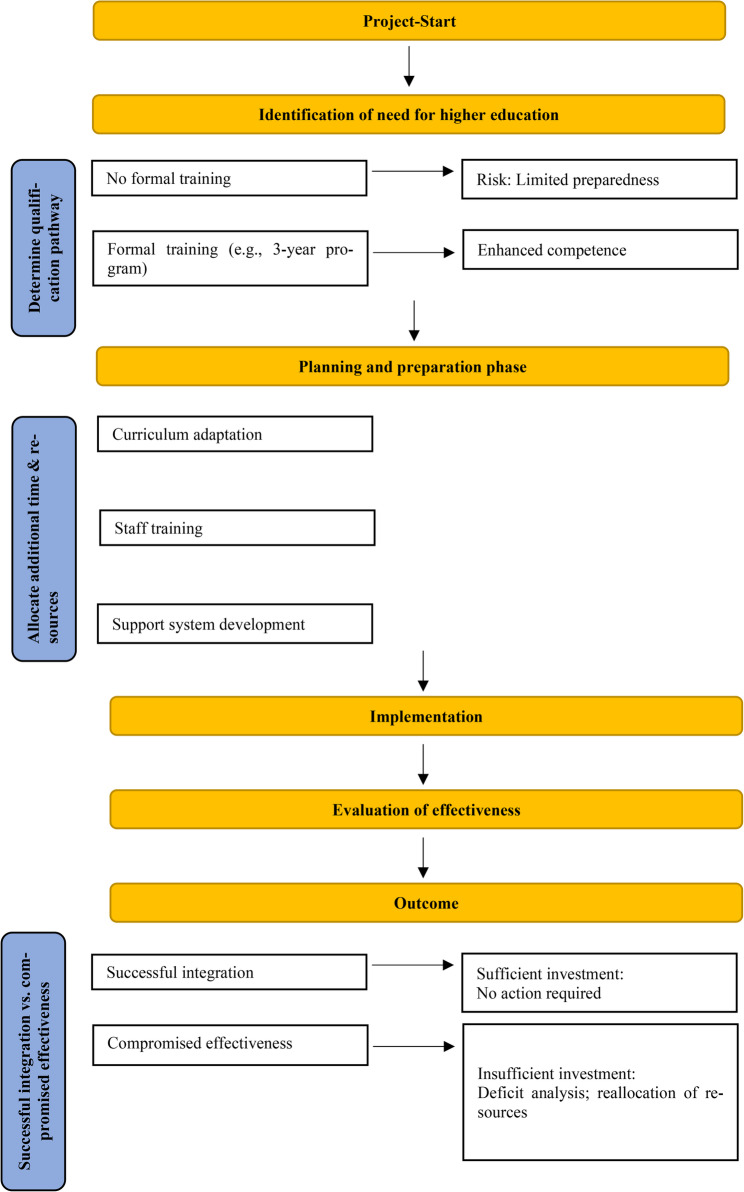
Schematic overview of the planning, implementation, and evaluation of co-teaching initiatives with individuals with Disorders of Intellectual Development in higher education and medical training, highlighting qualification of co-teachers, their integration into teaching activities, and the dependence of successful outcomes on adequate institutional resources and support

Figure 3 presents a flow diagram of the planning process for co-teacher qualifications, highlighting the importance of resource allocation and its potential impact on the success of outcomes, as synthesized from the experiences and findings reported in the reviewed literature.

### Mode of integration of co-teachers

A guideline article [22] described a structured three-year qualification program featuring modules on work and education, participation, educational practice, teaching methods, and assessment. This guideline emphasized the importance of varied instructional strategies, including repetition and practice, use of visual support, establishment of routines, prioritization of group work, creation of worksheets over didactic instruction, use of clear and accessible language, flexibility, creativity, and fostering an enjoyable learning environment.

Analysis of the implementation modalities revealed that co-teachers most frequently led colloquial seminars or individual course sessions, such as communication skills seminars [24, 25, 27]. In some studies, co-teachers were supported by assistants providing technical or general support [25, 27]. In other scenarios, co-teachers collaborated with academic staff to deliver between 12 and 16 two-hour lectures and actively participated in planning course modules [25, 27]. The presence of a support person was underscored as crucial for classroom management and reassurance [30]. Although institutional involvement of individuals with Disorders of Intellectual Development was documented, further details were not specified.

Review data indicated that approximately half of interventions featured direct interaction between students and people with Disorders of Intellectual Development, however, only a small proportion (16%) featured individuals with Disorders of Intellectual Development as co-teachers [31]. Most courses were offered as electives, and several authors emphasized the necessity for involvement of individuals with Disorders of Intellectual Development in roles beyond teaching alone [6]. Co-teaching models varied, including one teach/one assist and team teaching, while collaborative planning and feedback were consistently identified as crucial to effective implementation.

Reviews of user involvement in social care across England and Wales identified key barriers, including exclusionary system structures, institutional practices, and prevailing professional attitudes. These structural impediments undermined the transformative potential impact on student learning that could result from the active participation of individuals with Disorders of Intellectual Development [29]. Table 3 summarizes the essential strategies and supports for effective, inclusive co-teaching, as recommended by the reviewed literature and best practice guidelines.

**Table 3:** Recommended Instructional Strategies for Inclusive Co-Teaching

Strategy/Measure	Explanation/Example
Repetition & Practice	Reinforce key concepts through repeated, hands-on learning
Visual Supports	Incorporate diagrams, charts, and other visual aids to clarify content.
Routines	Establish consistent classroom routines to promote structure.
Group Work	Prioritize collaborative learning through structured group activities.
Worksheets	Use worksheets instead of lectures
Easy Language	Employ clear, jargon-free, and accessible language for all instruction.
Flexibility	Adapt instructional approaches to address individual needs.
Creativity	Employ varied and innovative teaching methods to enhance engagement.
Enjoyable Environment	Foster a positive, supportive classroom atmosphere
Support Persons	Provide additional personnel for classroom management and reassurance.
Collaborative Planning	Engage co-teachers in curriculum development and feedback processes
Joint Lectures	Facilitate delivery of lectures by co-teachers and academic staff.
Involvement Beyond Teaching	Engage Individuals with Disorders of Intellectual Development in multiple roles beyond instructional duties

### Source: author’s work

#### Experiences by co-teachers during Implementation

Two studies [28, 30] reported on co-teachers’ perspectives during implementation. Mevold et al. [30] reported that co-teachers initially felt discomfort, particularly during their first lectures and when confronted with questions considered overly personal or invasive. In addition to the described challenges, central themes reported were a high perceived degree of autonomy at work; a positive perception of new relationships; and perception of accomplishment. In Rodríguez Herrero et al. [28], co-teachers reported to have no formal training in teaching. One co-teacher highlighted challenges with topics less connected to their personal experiences, stating that sessions on Universal Design for Learning were particularly challenging. Further specific training needs were recognized in assessment, classroom management and public speaking. Another challenge reported was the grading of students, particularly those without disabilities [28]. The study identified essential prerequisites for co-teacher implementation, including the development of abilities (such as basic teaching methods, presentation skills and assessment strategies) both pre-service and in-service in order to improve the practice of co-teacher implementation. Two articles [24, 25] highlighted the necessity of preparatory meetings prior to teaching activities, especially for co-teachers with profound and multiple disabilities and Disorders of Intellectual Development. Effective inclusive involvement required robust support, guidance, and mentoring within a partnership model that respected the expertise and autonomy of individuals with Disorders of Intellectual Development, enabling their control over participation and shared content.

### Impact on students

Most studies evaluated the effects of co-teaching on students. Six articles [6, 25, 27–29, 31] reported enhanced student awareness of specific communication challenges following co-teaching sessions.

The improvements followed lectures led by formally trained educational specialists with Disorders of Intellectual Development and were described by students as changes in attitude toward inclusive education, as well as self-efficacy expectations. As part of the seminar curriculum, lived experiences were shared by the educational specialists. Among student-participants, 56% reported a change in their attitude toward inclusive education post-intervention. Students also demonstrated a high overall level of instructional self-efficacy. However, improvements in both domains were limited to select items on the respective assessment scales [24].

Mechler et al. [27] focused on the effects of lessons delivered and extracurricular contributions by educational specialists on students. Findings were categorized into three domains: interpersonal skills (e.g., perspective-taking), perceptions of inclusion and individuals with Disorders of Intellectual Development (e.g., self-reflection), and professional knowledge (e.g., behavioural intentions as future teachers). Although some participants viewed the intervention as not effective (e.g. redundancies with existing curriculum contents and lack of concrete guidelines for sustainable change), many others reported multifaceted and positive changes across all domains.

Four studies [25–27, 31] demonstrated increased student confidence and comfort in interacting with individuals with Disorders of Intellectual Development. Direct personal contact with people with Disorders of Intellectual Development proved more beneficial than indirect teaching methods [31]. One study [29] noted improved information retention, attributed to increased salience. Although some students reported initial difficulties engaging with individuals with Disorders of Intellectual Development [26] or challenges adopting person-centered or group-oriented perspectives [27]. Additionally, exposure to individuals with Disorders of Intellectual Development prompted students to recognize and reflect on their personal biases regarding disability and inclusion [28]. Tracy et al. [25] observed modest knowledge gains and a statistically significant but small reduction in self-reported discomfort following a single 3-hour communication session, particularly among students without prior experience with individuals with disorders of intellectual development. Although IDP scores decreased significantly in 128 fourth-year students (73.7 to 70.41; *t*(127) = 5.07, *p* < .001), the effect size was small (~ 0.3 *SD*), outcomes were based on self-report immediately post-intervention, and reliability and factor structure were not reassessed in this sample.

### Impact on co-teachers

One study specifically examined the effects of inclusive teaching activities on co-teachers, demonstrating improvements in teaching abilities, social competence, and self-awareness [30]. The authors noted that these outcomes correspond to the three basic psychological needs: autonomy, competence, and relatedness. These were described in self-determination theory and considered fundamental for intrinsic motivation and well-being. A review [31] also highlighted the positive experiential value for co-teachers in having the opportunity to instruct students. Additional studies [26–29] reported enhanced empowerment and a greater sense of being heard among co-teachers, alongside expanded social networks and improved health status despite the role’s emotional demands [23].

### Impact on faculty

Rodríguez Herrero et al. [28] assessed the impact of co-teaching on academic instructors. The medical teachers reported personal and professional growth, including adoption of a more facilitative and less directive teaching style, recognition of alternative pedagogical approaches’ efficacy, and adaptation to grant co-teachers greater classroom autonomy. The co-teaching experience positively influenced the instructor’s conceptualization of inclusion, teaching identity, and overall professional development. The collaboration with co-teachers with Disorders of Intellectual Development contributed to a deeper understanding of inclusive education and highlighted the value of integrating diverse perspectives into teacher training. However, the study also concluded that the inclusive co-teaching model required further refinement to optimize benefits for all participants.

**Fig. 4:**
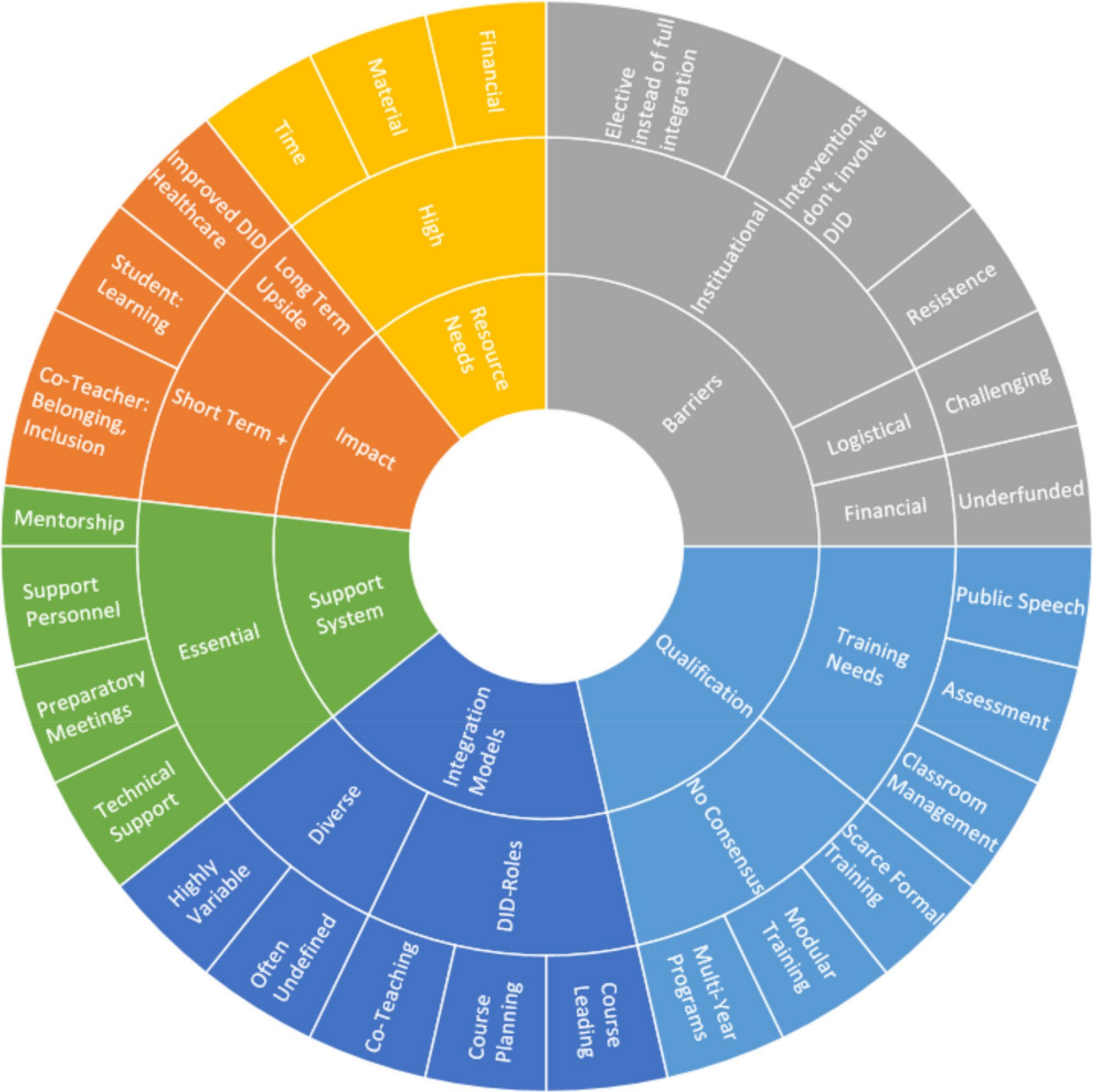
Legend: Thematic overview of factors influencing co-teaching initiatives with individuals with Disorders of Intellectual Development in higher education and medical training. The sunburst diagram depicts four main domains (qualification, integration models, support systems, and resource needs and impact)summarizing reported training requirements, role definitions, essential supports, barriers, and short- and long-term benefits for students, co-teachers, and healthcare

Meanwhile, Ward et al. [23] highlighted several important faculty experiences in designing and implementing inclusive teaching alongside a co-teacher with profound and multiple disabilities and Disorders of Intellectual Development. Faculty noted that collaborating with a co-teacher prompted critical reflection on their assumptions regarding ability and expertise. They assumed responsibility for logistical aspects, including ensuring classroom accessibility, orienting students to the distinctive teaching model, and adapting to unforeseen health or communication needs of the co-teacher. Effective inclusive teaching was characterized as reliant on strong collaboration, thorough preparation, and mutual trust among faculty, the co-teacher, and their support network. Faculty described the process as transformative for their professional growth, recognizing the emotional investment required to both support the co-teacher and maintain a meaningful, non-exploitative experience. Ultimately, faculty viewed this approach as pivotal for advancing inclusive education and social justice, while emphasizing the necessity for ongoing institutional self-examination, enhanced support mechanisms, and broader participation of individuals with Disorders of Intellectual Development. Figure 4 provides a visually organized summary of this reviews’ key findings, organized by focal points.

## Discussion

Based on the findings reported across studies, the implementation of inclusive co-teaching involving individuals with Disorders of Intellectual Development indicates multidimensional benefits, both pedagogical and personal, for all stakeholders, while simultaneously highlighting areas in need of further development and refinement.

The majority of studies reviewed focused on student outcomes and consistently reported positive effects on learners. Students participating in courses co-taught by individuals with Disorders of Intellectual Development demonstrated increased awareness of communication challenges [6, 27–29, 31], improved attitudes toward inclusive education and heightened self-efficacy expectations, with some studies showing improvements, albeit limited to specific assessment items [24]. Furthermore, interpersonal skills, such as empathy and perspective taking improved, self-reflection was enhanced, and professional intentions were influenced [27]. Higher levels of comfort and confidence in interacting with individuals with Disorders of Intellectual Development were observed [25–27, 31], with direct contact proving notably more effective than indirect methods [31]. A beneficial impact on knowledge acquisition and retention, especially regarding person-centered communication and practice was also evident [25]. These outcomes reflect that co-teaching not only improves cognitive learning but also fosters affective and attitudinal development in students; skills essential for future educators and healthcare professionals working toward inclusive practice.

Although fewer studies investigated the outcomes for co-teachers with Disorders of Intellectual Development, the available findings highlight a number of substantial personal and professional benefits. Mevold et al. [30] demonstrated how participation in inclusive teaching enhanced co-teachers’ teaching skills, social competence, and self-awareness. These outcomes aligned with the three essential psychological needs defined by Self-Determination Theory; autonomy, competence, and relatedness, which are considered foundational for intrinsic motivation and psychological well-being. Similarly, a review by Shakespeare and Kleine [31] affirmed the experiential value of co-teaching, emphasizing the significance of this instructional role for personal growth. Other studies [26, 28, 29] reported heightened feelings of empowerment and validation among co-teachers, who expressed a strengthened sense of being heard and acknowledged. These experiences were also associated with broader social benefits, including expanded networks and improved well-being. Despite these positive outcomes, the emotional demands of the role were acknowledged, particularly regarding public speaking and engagement with unfamiliar content [23]. Rodríguez Herrero et al. [28] identified several key challenges faced by co-teachers, including a lack of formal pedagogical training and difficulties delivering content unrelated to personal experiences, for example in teaching universal design for learning. Additional training needs were identified, especially in areas such as assessment strategies, classroom management, and public speaking. The study emphasized the importance of both pre-service and in-service training to support these competencies and strengthen the overall implementation of co-teaching models. The transition from initial discomfort to feelings of competence and accomplishment highlights the importance of comprehensive training, mentorship, and partnership models that respect individual autonomy and provide need-based support.

Only one study [28] directly examined the impact of inclusive co-teaching on academic staff. The involved instructor reported relevant professional and personal development, including a shift toward a more facilitative and less directive teaching style. The experience revealed the effectiveness of alternative pedagogical approaches and encouraged the instructor to provide co-teachers with increased autonomy in designing and delivering sessions. Participation in co-teaching reshaped the instructor’s understanding of inclusion and contributed to the evolution of their teaching identity and overall professional development. Moreover, collaboration with co-teachers with Disorders of Intellectual Development deepened the instructor’s awareness of inclusive education and highlighted the value of integrating diverse lived experiences into educational settings. However, the study also acknowledged that the inclusive co-teaching model remained in need of further refinement. For this model to reach its full potential and maximize benefits for all participants, additional structural and pedagogical adjustments were recommended.

The inconsistent terminology prevalent in German-speaking regions, such as intellectual disability, developmental disability, developmental disorder, and learning disability, presents an additional challenge for research and development in this field, as relevant knowledge and practical experience overlooked due to insufficient or imprecise search terms. The lack of German-language scientific literature on this subject is particularly striking, especially given the repeated calls for more inclusive educational practices in national CRPD monitoring reports (2015, 2023).

To address the research question and provide as comprehensive an overview as possible, this review included studies irrespective of statistical significance, reflecting the current scarcity of research on the involvement of individuals with Disorders of Intellectual Development as co-teachers. The primary aim was not to appraise study quality, but rather to capture and synthesize the breadth of available evidence to inform future research and practice.

The findings of this review align with the heterogeneous CRPD implementations outlined in the introduction, Germany at 35th globally versus established UK service user involvement in health professions education. Molnar et al. [8] synthesise current WHO and UN guidance and highlight that disability-inclusive, co-produced training should become a routine part of accreditation standards and continuing professional development; our practice points align with this agenda by specifying how co-teaching can be embedded within health curricula [32]. The scarcity of robust German-language evaluations positions co-teaching as both pedagogic advance and systemic benchmark, necessitating standardized, resourced models in medical curricula to meet legal mandates and equitable training goals.

## Conclusions

The collective findings from the reviewed literature highlight the potential and multifaceted benefits of inclusive co-teaching models involving individuals with Disorders of Intellectual Development. Evidence demonstrates that such models seem to enhance student awareness, empathy, and confidence in working with people with Disorders of Intellectual Development, as well as improve knowledge acquisition and retention. For co-teachers with Disorders of Intellectual Development, participation in instructional roles may foster personal growth, empowerment, and social connection, while contributing positively to well-being and professional identity. Academic instructors, though only assessed in one study, also reported personal and professional transformation, including the adoption of more inclusive pedagogical approaches and a greater understanding of diversity in educational contexts.

At the same time, the literature underscores important challenges that must be addressed to optimize the impact and sustainability of co-teaching initiatives. These include the need for structured and ongoing training for all co-teachers, comprehensive support systems within educational settings, and deliberate attention to role definition and collaborative planning. Emotional demands, especially for co-teachers with Disorders of Intellectual Development, and the need for further refinement of partnership models are recurring themes that warrant continued focus.

Overall, inclusive co-teaching emerges as a promising approach for advancing not only educational goals but also broader equity and inclusion. With appropriate investment in training, support, and institutional commitment, the co-teaching model offers a pathway for transforming educational practice and empowering all participants (students, co-teachers, and academic staff alike).

Current best practices and pilot initiatives in German higher education illustrate recent developments in inclusive teaching. Examples include social studies programs [33] and educational science projects [34], both of which showcase innovative approaches to involving individuals with Disorders of Intellectual Development in higher education. At our institution, the FRiMeL project [33–35], *Research Project for the Implementation of Inclusive teaching in Medicine*, aims to develop a training curriculum designed to prepare individuals with Disorders of Intellectual Development to serve as co-teachers in medical education for university students.

While these efforts signal progress, broader and more lasting impact will depend on continued research, comprehensive documentation, and the dissemination of data regarding practical experiences, challenges, and effective strategies. Only through such sustained efforts, structural changes can be implemented to reduce systemic barriers and fulfill the legal right to equality for individuals with Disorders of Intellectual Development.

### Practice points

Based on the reviewed literature, key practice points have been synthesized to serve as recommended actions aimed at addressing the current gaps in scientific evidence, developing evidence-based qualification frameworks, and supporting implementation across various educational disciplines.

- Co-teaching with individuals with Disorders of Intellectual Development seems to improve empathy in healthcare students.

Integrate individuals with Disorders of Intellectual Development as co-teachers within healthcare curricula to foster student empathy and reduce stigma through exposure to lived experiences [25–27, 31].

- Training and support are vital for effective co-educator participation.

Provide structured, ongoing training to both, faculty and co-teachers with Disorders of Intellectual Development, emphasizing instructional competencies, classroom management, and adaptive, evidence-based pedagogy [23, 28].

- Institutional backing ensures sustainability of inclusive programs.

Ensure institutional commitment by securing leadership support, transparent outcome reporting, and mandating inclusive co-teaching within policy and resource allocation to optimize student and faculty benefits [6, 24, 25, 27–31].

- Standardized guidelines are needed for consistent implementation.

Establish and apply evidence-based, standardized guidelines for co-teaching, utilizing universal design for learning to maintain instructional quality and consistency [27].

- Research and policy must address barriers to inclusion.

Systematically identify and address institutional barriers, such as language, recruitment, and accessibility, while promoting interdisciplinary collaboration for sustained innovation in inclusive education. Standardized guidelines for implementation should also be developed based on best practices in universal design for learning [1, 2, 33–35].

### Limitations

Several limitations should be considered when interpreting these findings. Most notably, the existing literature predominantly describes interventions implemented within elective courses. As a result, the reported outcomes reflect learning environments in which students had already chosen to engage with inclusion-related content and were often highly motivated to participate. This self-selection may overestimate positive attitudinal or experiential effects and limits the generalisability of the findings to settings where participation is mandatory and student motivation is more heterogeneous. This focus limits the generalizability and potential impact of inclusive educational models, especially in disciplines such as medicine, where future practitioners will inevitably interact with individuals with Disorders of Intellectual Development. Expanding inclusive teaching to mandatory coursework could not only reduce apprehension towards contact, but also promote communication competencies and support more sustainable, society-wide integration efforts. Incorporating such models into compulsory curricula would also provide greater opportunities for rigorous study designs, systematic evaluations, and interdisciplinary research initiatives.

In addition, elective offerings are typically more flexible in terms of group size, staffing, and scheduling, and they may benefit from committed local champions. These conditions are not always replicable in core curricula, where timetabling constraints, assessment requirements, and competing content demand more standardised structures. Consequently, the evidence summarised in this review provides important proof-of-concept indications but does not yet demonstrate that co-teaching with individuals with Disorders of Intellectual Development can be sustained at scale within required programmes. Future studies should therefore explicitly examine implementation in compulsory modules, including organisational prerequisites, resource implications, and the experiences of students who have not self-selected into such courses.

Additional limitations include the variation in terminology across regions, which may have led to relevant studies being overlooked due to inconsistent search terms. Furthermore, many included studies relied on self-reported outcomes or qualitative data, often lacking long-term follow-up. There is also limited representation of interventions from non-English-speaking and non-Western contexts, restricting the global applicability of the results. Lastly, the scarcity of research on the perspectives and effects for faculty members, as well as on large-scale, system-level outcomes, highlights areas in need of further investigation.

Further, the qualitative thematic synthesis, driven by six a priori analytic categories, may have precluded meta-analysis due to heterogeneous outcomes, potentially limiting generalizability. Quantitative outcome data were often reported in a limited way, with some studies providing only descriptive statistics or narrative statements without inferential tests. This restricts the precision with which effect sizes and the strength of quantitative evidence can be assessed in this review.

Analytic decisions to incorporate two non-empirical project reports and one guideline alongside seven primary studies enriched contextual insights but introduced methodological variability, underscoring the need for standardized quality appraisal of included interventions in future reviews.

## Data Availability

Data sharing is not applicable to this article as no new datasets were generated. All materials analyzed during this study are included in the published article and its supplementary information files.

## Abbreviations

FRiMeL: Forschungsprojekt Zur Realisierung Inklusiver Medizinischer Lehre
